# Effect and safety of dual anti-human epidermal growth factor receptor 2 therapy compared to monotherapy in patients with human epidermal growth factor receptor 2-positive breast cancer: a systematic review

**DOI:** 10.1186/1471-2407-14-625

**Published:** 2014-08-28

**Authors:** Xiao Zhang, Xin-Ji Zhang, Tian-Yi Zhang, Fei-Fei Yu, Xin Wei, Ye-Sheng Li, Jia He

**Affiliations:** Department of Health Statistics, Second Military Medical University, Shanghai, China; Renji Hospital, Shanghai Jiao Tong University School of Medicine, Shanghai, China; Department of Special Treatment, Eastern Hepatobiliary Hospital, Second Military Medical University, Shanghai, China

**Keywords:** anti-HER2 therapy, HER2-positive breast cancer, Systematic review

## Abstract

**Background:**

Dual anti-human epidermal growth factor receptor 2 (HER2) therapies have been shown to improve outcomes of HER2-positive breast cancer patients. We undertook a systematic review to compare treatment outcomes for patients who received single or combined anti-HER2 therapies.

**Methods:**

We identified randomized control trials that compared dual anti-HER2 therapy and anti-HER2 monotherapy in patients with HER2-positive breast cancer. Outcomes included pathologic complete response (pCR), overall survival (OS), progression-free survival (PFS), and adverse events. Included in the analysis were seven trials that recruited 2,609 patients.

**Results:**

In the neoadjuvant setting, the pooled pCR rate in the dual anti-HER2 therapy and monotherapy groups in combination with chemotherapy was 54.8% and 36%, respectively. This difference was statistically significant (relative risk, 1.56; 95% confidence interval (CI), 1.23–1.97; p < 0.001). In the metastatic setting, dual anti-HER2 therapy demonstrated significant benefits in both PFS (hazard ratio (HR), 0.71; 95% CI, 0.62–0.81; p < 0.001) and OS (HR, 0.68; 95% CI, 0.57–0.82; p < 0.001). Subgroup analyses indicated that the addition of chemotherapy to dual anti-HER2 therapy could greatly improve pCR in the neoadjuvant settings. However, in the metastatic setting, similar PFS and OS were found in patients receiving dual anti-HER2 therapy with or without chemotherapy. Dual anti-HER2 therapy was associated with more frequent adverse events than monotherapy, but no statistical differences were observed in cardiac toxicity.

**Conclusions:**

This systematic review provides a summary of all the data currently available, and confirms the benefits and risks of dual anti-HER2 therapy for HER2-positive breast cancer.

## Background

Despite advances in early diagnosis and treatment, breast cancer remains a significant public health concern; more than a million new cases are diagnosed each year, resulting in 400,000 deaths worldwide [1–3]. Human epidermal growth factor receptor 2 (HER2) protein is overexpressed in 15–20% of all breast cancers (HER2-positive breast cancer) and is associated with a poor outcome [4, 5].

The development of trastuzumab, a monoclonal antibody against HER2, has dramatically changed the prognosis for HER2-positive breast cancer patients [6]. Multiple randomized controlled trials (RCTs) have shown that trastuzumab therapy improves patient outcomes, and consequently trastuzumab has become the standard treatment for HER2-positive breast cancer patients in both the neoadjuvant and metastatic settings [7, 8]. Following trastuzumab, lapatinib, an anti-HER2 tyrosine kinase inhibitor, was approved for use, in combination with capecitabine, for the treatment of HER2-positive metastatic breast cancer that has progressed with standard treatment [9, 10].

Despite these improvements, resistance to these drugs remains a challenge, and novel therapeutic approaches are required [11, 12]. The effect of combining different anti-HER2-targeted agents is one therapeutic strategy currently under investigation [13]. Laboratory studies have shown that dual anti-HER2 therapy can block the signaling from HER2 and its related HER family members more completely, leading to increased cell death and tumor shrinkage in HER2-positive models of breast cancer [14]. The addition of pertuzumab, an anti-HER2 monoclonal antibody, to trastuzumab and docetaxel therapy significantly increases progression-free survival (PFS) for patients with HER2-positive metastatic breast cancer (median PFS, 19.5 versus 12.4 months) [15]. These impressive results have provided a strong rationale for conducting randomized controlled studies evaluating trastuzumab in combination with lapatinib or pertuzumab for HER2-positive breast cancer in both the adjuvant and metastatic settings. In this study, we conduct a systematic review of these RCTs to summarize the benefits and risks of dual anti-HER2 therapy, as compared with monotherapy, for HER2-positive breast cancer patients.

## Methods

### Data sources, search strategy, and selection criteria

The systematic review was performed according to the Quality of Reporting of Meta-analyses (QUORUM) guidelines [16]. We systematic searched PubMed, EmBase, MEDLINE, and the Cochrane Central Registered Controlled Trials for studies conducted prior to May 2013, using the following keywords: trastuzumab, pertuzumab, lapatinib, and breast cancer. The search was limited to randomized clinical trials, but without language restrictions. In addition, the American Society of Clinical Oncology (ASCO) Annual Meeting proceedings and the San Antonio Breast Cancer Symposium Meeting abstracts from 2004 to 2013 were individually searched for relevant randomized clinical trials. An independent search of relevant reviews and meta-analyses regarding trastuzumab, pertuzumab, or lapatinib was also conducted to ensure that no studies were missed. We reviewed each publication, and only the most recent or complete report of clinical trials was included when duplicate publications were identified. Efforts were also made to contact the study authors when relevant data were not clear.

The goal of this study was to evaluate the efficacy and safety of dual anti-HER2 therapy compared to monotherapy. Thus, only RCTs comparing dual anti-HER2 therapy (lapatinib, trastuzumab, or pertuzumab) with anti-HER2 monotherapy, with or without chemotherapy, in breast cancer were eligible. Phase I trials and single-arm phase II trials were excluded because of an absence of controls. Eligible studies had to meet the following inclusion criteria: studies had to 1) be prospective phase II and III trials with HER2 breast cancer patients, and 2) assign patients randomly to anti-HER2 single agent or combination treatment. The literature search and selection were undertaken independently by two investigators (Xiao Zhang and Fei-Fei Yu), and any disagreement was investigated by a third investigator (Xin-Ji Zhang) until a consensus was reached.

### Data extraction and quality assessment

Data extraction and collection was performed independently by two reviewers (Xiao Zhang and Fei-Fei Yu) using a standardized protocol. Disagreements were adjudicated by a third reviewer (Xin-Ji Zhang) after referring to the original articles. The following information was extracted from each included study for baseline characteristics: the first author’s surname, publication year, original country, age, number of patients per arm, dose and duration of anti-HER2 therapy, type/dose of chemotherapy, median follow-up period, and outcome measures. End points of interest included overall survival (OS), PFS, pathologic complete response (pCR), and adverse events (AEs). The quality of the trials included in this study were assessed using the Jadad scale [17]. The trials were assessed on the basis of randomization, concealment of treatment allocation, blinding, completeness of follow-up, and the use of intention-to-treat analysis.

### Statistical analysis

We extracted the adjusted hazard ratio (HR) and corresponding 95% confidence interval (CI) from each RCT to estimate the pooled HR, and corresponding 95% CI, for OS and PFS in the dual anti-HER2 therapy and monotherapy groups. We also allocated dichotomous frequency data to assess the pooled relative risks (RRs), and corresponding 95% CI, of pCR and each AEs in the dual anti-HER2 therapy and monotherapy groups. Included in the analysis were five three-arm trials, with two anti-HER2 monotherapy arms and one dual anti-HER2 therapy study. The two anti-HER2 monotherapy arms were merged into one group by adding the sample size and the number of events in each arm. Subgroup analyses were performed according to the addition of chemotherapy and the component drugs of dual anti-HER2 therapy (trastuzumab + lapatinib or trastuzumab + pertuzumab). When there was no statistically significant heterogeneity, a pooled effect was calculated using a fixed-effect model; otherwise, a random-effect model was employed. Heterogeneity of treatment effects between trials was evaluated using the chi-square (χ^2^) test and I-squared (I^2^) statistic [18]. We also assessed the probability of publication bias using the Egger’s [19] and Begg–Mazumdar [20] tests. Statistical analyses were carried out using STATA 11.0 (State Corporation, Lake Way, Texas, USA). A two-tailed p value <0.05 was considered statistically significant.

## Results

### Search results and trial characteristics

**Figure 1:**
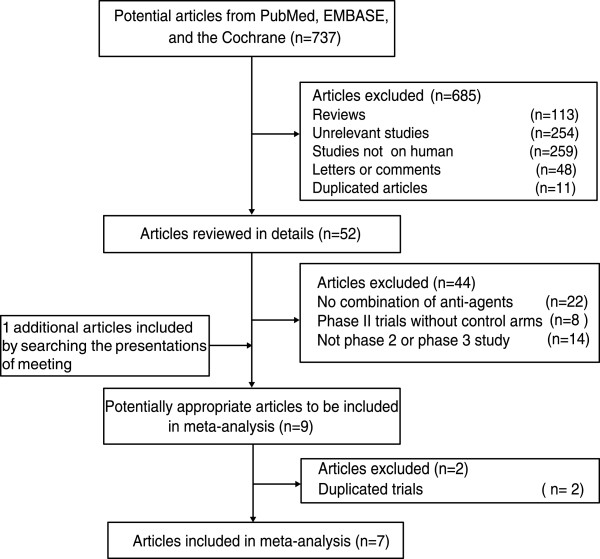
**Flow diagram of the trial search and selection process.**

We identified 737 potentially relevant trials from our initial electronic search, and excluded 685 trials after a preliminary review. The remaining 52 studies were retrieved for detailed assessment. Of these, 44 articles were excluded because they contained no combination therapy group, were phase II trials without a control arm, or they were not phase II or phase III studies. An additional article was included by searching ASCO 2012 Annual Meeting abstracts [21]. A further two studies were excluded because they were duplicates [22, 23]; only the most recent reports were included [15, 24]. Therefore, a total of seven [15, 21, 24–28] RCTs were included in our final analysis, which included data on 2,609 individual patients (Figure 1). The design characteristics of the included trials are presented in Table 1. The role of dual anti-HER2 therapy in the neoadjuvant setting was evaluated in five trials [21, 25–28] and two trials [15, 24] investigated dual anti-HER2 therapy in the metastatic setting. All the breast cancer patients were HER2-positive. The quality of the trials was assessed according to the pre-determined criteria of the Jadad score. Overall, three trials had a Jadad score of 5 [23, 26, 27], three trials had a score of 4 [21, 25, 28], and one trial had a score of 3 [24].

**Table 1:** **Character of the included studies**

Study	Phase	Treatment staus	Treatment arms	Pts no.	Dosage	Chemotherapy	Treatment duration	Median age	Jada score	Follow- up (m)
CHER-LOB^26^ (2012)	2	Neoadjuvant	T	36	2 mg/kg weekly (loading 4 mg/kg)	Paclitaxel	26 weeks	50	5	NA
			L	39	1500 mg daily	Paclitaxel	26 weeks	49		NA
			L + T	46	1000 mg daily + 2 mg/kg weekly (loading 4 mg/kg)	Paclitaxel	26 weeks	49		NA
NeoALTTO^27^ (2012)	3	Neoadjuvant	L	149	1500 mg daily	Paclitaxel	18 weeks	50	5	NA
			T	154	2 mg/kg weekly (loading 4 mg/kg)	Paclitaxel	18 weeks	49		NA
			L + T	152	1000 mg daily + 2 mg/kg weekly (loading 4 mg/kg)	Paclitaxel	18 weeks	50		NA
NSABP B-41^21^ (2012)	3	Neoadjuvant	T	177	2 mg/kg weekly (loading 4 mg/kg)	Paclitaxel	12 weeks	NA	4	NA
			L	171	1250 mg daily	Paclitaxel	13 weeks	NA		NA
			L + T	171	750 mg daily + 2 mg/kg weekly (loading 4 mg/kg)	Paclitaxel	14 weeks	NA		NA
NeoSphere^25^ (2012)	2	Neoadjuvant	T	107	6 mg/kg q3w (loading 8 mg/kg)	Docetaxel	12 weeks	50	4	NA
			T + P	107	6 mg/kg q3w (loading 8 mg/kg) + 420 mg/kg q3w (loading 840 mg/kg)	docetaxel	13 weeks	50		NA
			T + P	107	6 mg/kg q3w (loading 8 mg/kg) +420 mg/kg q3w(loading 840 mg/kg)	NO	14 weeks	49		NA
			P	96	420 mg/kg q3w (loading 840 mg/kg)	docetaxel	15 weeks	49	4	NA
Holmes^28^ (2012)	NA	Neoadjuvant	T	33	2 mg/kg weekly (loading 4 mg/kg)	FEC	26 weeks	NA		NA
			L	34	1250 mg daily	FEC	27 weeks	NA		NA
			L + T	33	750 mg daily + 2 mg/kg weekly	FEC	28 weeks	NA		NA
EGF104900^24^ (2012)	3	Metastatic	L + T	148	1000 mg daily + 2 mg/kg weekly	No	Until progression or unacceptable toxicity	51	3	12.8
			L	148	1500 mg daily	No	Until progression or unacceptable toxicity	51		8.7
CLEOPATRA^15^ (2012)	3	Metastatic	P + T	402	6 mg/kg q3w (loading 8 mg/kg) + 420 mg/kg q3w (loading 840 mg/kg)	Docetaxel	Until progression or unacceptable toxicity	54	5	30
			Placebo + T	406	6 mg/kg q3w (loading 8 mg/kg)	Docetaxel	Until progression or unacceptable toxicity	54		30

*Abbreviations*: *T* Trastuzumab, *L* Lapatinib, *P* Pertuzumab, *pts no* patients number, *mg* milligram, *kg* kilogram, *Pacl* Paclitaxel, *FEC* Fluorouracil (5FU), epirubicin, and cyclophosphamide; *q3w* three-weekly, *NA* Not available, *m* month.

### pCR in neoadjuvant studies

**Figure 2:**
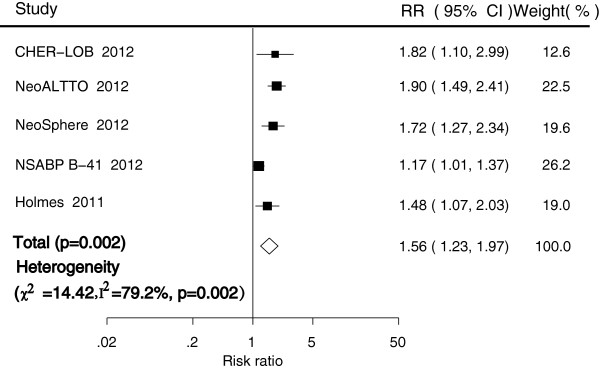
**Meta-analysis of pathologic complete response between dual anti-HER2 therapy and monotherapy groups.**

pCR was evaluated in a total of five trials [21, 25–28], all which investigated the effect of anti-HER2 therapy in the neoadjuvant settings. In these trials, anti-HER2 agents were combined with chemotherapy: paclitaxel in three trials [21, 26, 27], FEC in one trial [28], and docetaxel in the remaining one trial [25]. There was also one arm without chemotherapy in the Neo-Sphere trial [25], and this arm was excluded in the pooled analysis. The pooled pCR rate was 54.8% (278 of 508 patients; 95% CI, 0.46–0.63) in the dual therapy group compared with 35.6% (442 of 995 patients; 95% CI, 0.24–0.50) in the monotherapy group. The difference in pCR between dual agents and single anti-HER2 agents was significant (RR, 1.56; 95% CI, 1.23–1.97; p < 0.001), with no evidence of significant publication bias (Egger’s test, P = 0.624; Begg-Mazumdar test, P = 0.9370; Figure 2).We noted some evidence of heterogeneity in the magnitude of this effect across the included studies (p = 0.006, I^2^ = 72.3%), which was mostly attributable to inclusion of the NSABP B-41 study [21]. A sensitivity analysis that excluded the NSABP B-41 study resulted in a similar RR (1.74; 95% CI, 1.49–2.03; p < 0.001) with much reduced heterogeneity (p = 0.67, I^2^ = 0.00%).

### PFS and OS in metastatic studies or setting

In our analysis, two studies [15, 24] investigated the effect of dual anti-HER2 therapy in the metastatic setting. The CLEOPATRA study was a phase III study that included 808 patients with HER2-positive metastatic breast cancer [15]. In this study, patients were randomized to first-line treatment of pertuzumab + trastuzumab + docetaxel (dual anti-HER2 therapy group) or trastuzumab + docetaxel + placebo (control group). The median PFS was 18.7 months in the dual anti-HER2 therapy group and 12.4 months in the control group. The difference in PFS was significant (HR, 0.69; 95% CI, 0.58–0.81; P < 0.001). Furthermore, a significant benefit in OS was also observed in patients allocated to the dual anti-HER2 therapy treatment group compared with individuals assigned to the control group (HR, 0.66; 95% CI 0.52 - 0.84; p < 0.001). The median OS for patients allocated to the control group was 37.6 months but was not reached in the dual anti-HER2 therapy group. Another phase III study (EGF104900) enrolled patients with HER2-positive metastatic breast cancer whose disease had progressed during prior trastuzumab therapy [24]. In this study, 296 patients were randomly assigned to receive either lapatinib + trastuzumab (dual anti-HER2 therapy group) or lapatinib monotherapy (control group). The median PFS was 11.1 and 8.1 weeks in the dual anti-HER2 therapy and control groups, respectively. Median OS was 14 months for the dual anti-HER2 therapy group and 9.5 months for the lapatinib alone group. The dual anti-HER2 therapy group showed significant improvements in PFS (HR, 0.74; 95% CI, 0.58–0.94; P = 0.011) and OS (HR, 0.74; 95% CI, 0.57–0.97; P = 0.026). Although these two trials had different patient populations and settings, both showed a significant improvement in OS and PFS with dual anti-HER2 therapy.

### Subgroup analysis

Subgroup analyses were performed to determine whether the type of neoadjuvant chemotherapy influenced the efficacy of dual anti-HER2 therapy. We found that the benefit of dual anti-HER2 therapy on pCR was similar among different chemotherapies. Detailed information regarding these analyses is summarized in Table 2.

**Table 2:** **Subgroup analysis based on the type of chemotherapy and the component of dual agents**

Subgroup	Reference	pCR
The type of Chemotherapy	21,25,26,27,28	1.56 (1.23,1.97)
Paclitaxel	21,26,27	1.55 (1.06,2.28)
FEC	28	1.48 (1.07,2.03)
Docetaxel	25	1.72 (1.27,2.34)
Component of dual agents		
L + T	21,26,27,28	1.52 (1.15,2.01)
P + T	25	1.72 (1.27,2.34)

*Abbreviations*: *T* Trastuzumab, *L* Lapatinib, *P* Pertuzumab.

We also performed subgroup analyses based on the component drugs of dual anti-HER2 therapy (trastuzumab + lapatinib or trastuzumab + pertuzumab). The results of this subgroup analysis indicated that there were improvements in pCR in dual anti-HER2 therapies consisting of trastuzumab + lapatinib (RR, 1.52; 95% CI, 1.15–2.01) and trastuzumab + pertuzumab (RR, 1.72; 95% CI, 1.27–2.34; Table 2). Compared with monotherapy, dual anti-HER2 therapy consisting of trastuzumab + lapatinib showed a decrease in the HR for disease progression and death of 26% and 29%, respectively. Similar benefits in PFS (HR, 0.69; 95% CI, 0.58–0.81) and OS (HR, 0.66; 95% CI, 0.52–0.84) were also observed with trastuzumab + pertuzumab dual anti-HER2 therapy.

### Relative risk of AEs

**Figure 3:**
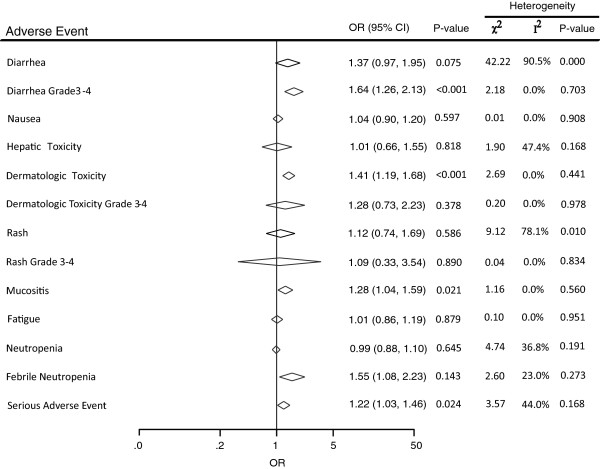
**Meta-analysis of adverse events between dual anti-HER2 therapy and monotherapy groups.**

The use of dual anti-HER2 therapy was associated with an increase in serious AEs (RR, 1.22; 95% CI, 1.03–1.46; p = 0.024). In addition, dual anti-HER2 therapy increased the risk of grade 3–4 diarrhea (RR, 1.64; 95% CI, 1.26–2.13; p < 0.001), all grades of dermatologic toxicity (RR, 1.41; 95% CI, 1.19–1.68; p < 0.001), and febrile neutropenia (RR, 1.55; 95% CI, 1.08–2.23; p = 0.143). No statistically significant differences were observed between the two arms for neutropenia (RR, 0.99; 95% CI, 0.88–1.10; p = 0.645), hepatic toxicity (RR, 1.01; 95% CI, 0.66–1.55; p = 0.818), grade 3-4 dermatologic toxicity (RR, 1.28; 95% CI, 0.73–2.23; p = 0.378), nausea (RR, 1.04; 95% CI, 0.90–1.20; p = 0.597), or fatigue (RR, 1.01; 95% CI, 0.86–1.19; p = 0.879; Figure 3).

### Cardiac toxicity

**Figure 4:**
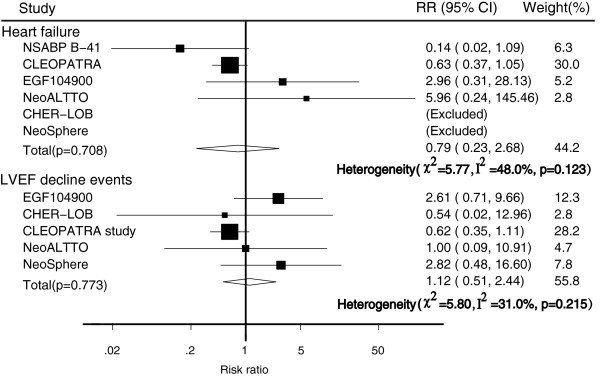
**Meta-analysis of cardiac toxicity between dual anti-HER2 therapy and monotherapy groups.**

The risk of heart failure was reported in six trials [15, 21, 24–27]. Overall, there was no statistically significant difference in the risk of heart failure between dual anti-HER2 therapy and monotherapy (RR, 0.79; 95% CI, 0.23–2.68; p = 0.708). There were five trials [15, 24–27] that reported data for left ventricular ejection fraction (LVEF) decline, but no statistically significant difference in risk between the treatment groups was found (RR, 1.12; 95% CI, 0.51–2.44; p = 0.773; Figure 4).

## Discussion

This systematic review summarizes all the available published, randomized evidence on the efficacy and safety of dual anti-HER2 therapy compared with monotherapy in HER2-positve breast cancer patients. Preclinical studies have suggested that combining different HER2 inhibitors with complementary mechanisms of action can help maximize the suppression of oncogenic processes involved in disease progression. Consequently, an increasing number of clinical trials have been performed to investigate this promising therapeutic approach [15, 24, 25]. Combining data from all the currently published trials, this systematic review confirms the benefit of dual HER2-directed therapy over monotherapy for HER2-positve breast cancer.

By pooling the data from the included trials, we showed that dual anti-HER2 therapy significantly improves pCR rate in the neoadjuvant setting (RR, 1.56; 95% CI, 1.23–1.97; p < 0.001). Hence, dual anti-HER2 therapy might be an attractive strategy in this setting. In the metastatic setting, dual anti-HER2 therapy, compared to monotherapy, demonstrated significant benefits for OS and PFS in the EGF104900 and CLEOPATRA studies. However, because of the limited data regarding metastatic disease, these benefits require further study.

Despite the advantages of dual anti-HER2 therapy in the neoadjuvant settings, a number of questions have arisen: 1) does the type of chemotherapy alter the efficacy of dual anti-HER2 therapy; and 2) which combination of the HER2 inhibitors has optimal efficacy for HER2-positive breast cancer. In this study, subgroup analyses were conducted to address these questions. When grouped by the type of chemotherapy, the improvement in the pCR rate with dual anti-HER2 therapy was similar, regardless of the specific chemotherapies used. Furthermore, similar efficacy was observed with trastuzumab + pertuzumab and trastuzumab + lapatinib. Based on current data, it appears that the type of chemotherapy and specific components of dual anti-HER2 therapy have little impact on the efficacy of the treatment. However, due to the limited number of trials in each subgroup, these results need to be validated in further studies.

Our study also raises some safety concerns. As the HER2 signaling pathway plays an important role in cardiac physiology [29], we investigated the risk of cardiac AEs. We found that no increased risk of cardiac toxicity was associated with dual anti-HER2 therapy, which is consistent with the results of a previous study conducted by Valachis et al. [30]. However, we found that some other toxicities were more frequent in patients who received dual anti-HER2 therapy, particularly grade 3–4 diarrhea, dermatologic toxicity, mucosis, febrile neutropenia, and other serious AEs. Clinicians should take note of this, since the incidence of AEs substantially affects a patient’s quality of life, and may lead to discontinuation of dual treatment.

Several limitations of this systematic review should be acknowledged. Firstly, data from the ongoing ALTTO and MARIANNE [14] trials are still unavailable. Secondly, our results are based on published data and presented clinical trials; individual patient data were not unavailable. Thirdly, because the results of this meta-analysis are confined to trastuzumab + lapatinib or trastuzumab + pertuzumab, the results are not necessarily applicable to treatments using other drug combinations. In addition, some subgroup analyses were based on limited studies, and the results should be interpreted with caution.

## Conclusions

This systematic review shows that, according to the present available data, dual anti-HER2 therapy seems to be more effective than monotherapy in the neoadjuvant setting. In the metastatic setting, limited data is available and further evaluation of the role of dual anti-HER2 treatment is required. However, it is reasonable to believe that a shift towards a dual therapeutic approach will yield clinically meaningful improvements for patients with HER2-postive breast cancer. However, given the increased risk of AEs associated with dual anti-HER2 therapy, it is important for health care practitioners to be aware of the potential risks and to provide close monitoring to improve patient outcome.
